# Gestational Trophoblastic Disease-induced Thyroid Storm

**DOI:** 10.5811/cpcem.2019.9.43656

**Published:** 2019-10-21

**Authors:** Carly Blick, Kraftin E. Schreyer

**Affiliations:** Temple University Hospital, Department of Emergency Medicine, Philadelphia, Pennsylvania

## Abstract

In the United States, gestational trophoblastic diseases (GTD), including molar pregnancies, occur in 121 out of 100,000 pregnancies.^1^ Many patients with GTD may develop hyperthyroidism. GTD-induced thyroid storm is a rare but life-threatening complication of GTD.^2^ Once patients are hemodynamically stable, the mainstay of definitive treatment is evacuation of the mole.^3^ We present a case of molar pregnancy-induced thyroid storm presenting as vaginal bleeding, fever, and tachycardia.

## INTRODUCTION

Vaginal bleeding at 6–16 weeks gestation is the most common presentation of hydatidiform moles.^1^ Molar pregnancy-induced hyperthyroidism is a rare but potentially life-threatening condition. Presentations can range from subclinical hyperthyroidism to thyrotoxicosis and thyroid storm. We describe a case of a patient with thyroid storm induced by molar pregnancy.

## CASE REPORT

A 39-year-old gravida 7 para 3033 female, with a history of three prior therapeutic abortions, presented to our emergency department (ED) with seven hours of heavy vaginal bleeding and crampy, lower abdominal pain. The patient reported her last menstrual period was four weeks prior to presentation. She also complained of one week of non-specific symptoms including intermittent nausea, vomiting, malaise, nasal congestion, and non-bloody diarrhea. She was found to be febrile to 102.2 degrees Fahrenheit and had a heart rate of 137 beats per minute (bpm). Her blood pressure was 124/86 millimeters of mercury, and oxygen saturation was 99% on room air.

The patient had a positive urine human chorionic gonadotropin (hCG) test. Ultrasound showed an empty uterus (Image), but her quantitative hCG was 117,495 milli-international units per milliliter (m[IU]/mL). The obstetrics (OB) team was consulted and evaluated the patient in the ED, and agreed that there was concern primarily for molar pregnancy versus septic abortion.

The patient was persistently tachycardic (to a maximum heart rate of 150 bpm), despite fever control with acetaminophen and intravenous fluid resuscitation. Electrocardiogram and cardiac monitor showed sinus tachycardia. Her white blood cell count was 16.1 thousands per cubic millimeter, and her hemoglobin was 11.3 grams per deciliter (g/dL). Thyroid stimulating hormone (TSH) was then sent and resulted as 0.009 m[IU]/mL. Total thyroxine (T4) was 14.7, micrograms (ug)/dL and free T4 was 1.82 nanograms (ng)/dL (Table), making the clinical picture concerning for thyroid storm.

A Burch-Wartofsky score, used for early detection of thyroid storm, was calculated at 55, which was highly suggestive of thyroid storm. At this point, the intensive care unit (ICU) team was also consulted. The patient was treated with propranolol 1 milligram (mg) followed by an additional 2 mg dose, propylthiouracil (PTU) 500 mg and solumedrol 80 mg, with improvement in tachycardia to 110 bpm. She was then taken to the operating room with obstetrics (OB) for a dilation and evacuation. She was treated for possible septic abortion with cefoxitin 2 grams (g), doxycycline 100 mg and metronidazole 500 mg. After operative intervention, the patient was admitted to the ICU for continued care.

After the procedure, the patient’s fever, leukocytosis and vaginal bleeding resolved. Postoperatively, her hemoglobin dropped to 7.8 g/dL and then remained stable. Endocrinology was consulted and thought the abnormal TSH was likely secondary to gestational hyperthyroidism, but could not rule out thyroid storm; they recommended checking thyroid stimulating immunoglobulin (TSI) to evaluate for Graves disease and discontinuation of steroids and PTU. The patient was transferred to the floor on postoperative day (POD) one and discharged home on POD two. TSI was negative. Prior to discharge, she received medroxyprogesterone acetate for contraception.

Pathology ultimately showed chorionic villi with marked hydropic changes consistent with complete hydatidiform mole and marked trophoblastic hyperplasia with cytologic atypia. She was recommended to have repeat beta hCGs checked weekly until negative for three weeks, and then monthly for six months, as well as thyroid function tests every four to six weeks until normal. She followed up with OB on POD eight and repeat hCG was 632 m[lU]/mL and was negative when she followed up four months later. She was lost to follow-up for endocrine and subsequent OB visits.

## DISCUSSION

Gestational trophoblastic disease (GTD) results from the proliferation of placental trophoblast cells. In the United States, GTDs are associated with about 121 per 100,000 pregnancies.^1^ GTD includes hydatidiform moles (complete and partial), invasive moles, choriocarcinomas, and placental site trophoblastic tumors. Hydatidiform moles occur when abnormal fertilization results in proliferative trophoblastic tissues and vesicular swelling of placental villi; these changes lead to a characteristic “grapelike” appearance.^4^ Most complete moles are 46,XX due to the fertilization of an empty ovum by a duplicated haploid sperm or two sperm. Most partial moles are caused by the fertilization of a normal ovum by two sperm and result in a triploid (69,XXY) karyotype.

Vaginal bleeding at 6–16 weeks gestation is the most common presentation of complete hydatidiform moles, occurring in 80–90% of cases.^1^ Other common symptoms include uterine enlargement greater than expected for gestational age and hyperemesis. Patients have elevated beta-hCG levels compared to normal pregnancies (often>100,000 m[IU]/mL) and absent fetal heart tones. Many cases initially present as a suspected missed or incomplete abortion. On ultrasound, a complete mole has a “snowstorm” granular appearance, although only 30–50% of hydatidiform moles are visualized by ultrasound.^3^,^5^ Once the diagnosis is established, it is important to evaluate patients for medical complications, such as anemia, preeclampsia, and hyperthyroidism.

**CPC-EM Capsule**

What do we already know about this clinical entity?

*Patients with gestational trophoblastic disease (GTD) may develop hyperthyroidism*.

What makes this presentation of disease reportable?

*GTD-induced thyroid storm is a rare but life-threatening complication of GTD*.

What is the major learning point?

*GTD-induced thyroid storm should be considered in any female patient of childbearing age with signs and symptoms of thyrotoxicosis*.

How might this improve emergency medicine practice?

*A high level of suspicion may help with faster diagnosis and initiation of treatment in this potentially life-threatening condition*.

Hyperthyroidism has been reported in many patients with GTD. The beta subunit of β-hCG is structurally similar to TSH, allowing it to bind to the TSH receptor on thyroid follicular cells.^5^ Of patients with hydatidiform mole 25–64% have been reported to have increased thyroid function, but only about 5% have clinical signs of hyperthyroidism.^6^ It has been reported that β-hCG levels of greater than 200,000 m[IU]/ml sustained for several weeks are required to induce clinical hyperthyroidism.^6^ It is estimated that for every 10,000 m[lU]/ml increase in serum hCG, TSH decreases by 0.1 mlU/ml and free T4 increases by 0.1 ng/dL.^5^ In normal pregnancy, the elevated β-hCG concentration induces a weak hyperthyroid state. This is heightened in molar pregnancy due to higher concentrations of β-hCG. Additionally, the molecular variants of the β-hCG present in molar pregnancy have increased thyrotropic activity, likely due to its decreased sialic acid concentration.^7^ The degree of increased thyroid hormone varies based on the level of β-hCG, the amount of desialylation of the β-hCG, and the duration of the molar pregnancy.

Presentations can range from subclinical hyperthyroidism to thyrotoxicosis and thyroid storm. GTD-induced thyroid storm is a rare but life-threatening complication of GTD.^2^ Thyroid storm is highly lethal, with a mortality of 10–30%, associated with tachycardia, fever, agitation, and altered mental status.^8^,^9^ Patients are often profoundly tachycardic (>140 bpm), and have limited response to calcium-channel blockers, beta blockers, and intravenous fluids. A high level of suspicion and early diagnosis is crucial to prevent the complications of thyroid storm such as stroke, dysrhythmia, heart failure, rhabdomyolysis, liver dysfunction, and death.^4^

The emergent management of thyroid storm involves decreasing hormone synthesis and release, blocking the action of thyroid hormone, reversing systemic decompensation, and removing the precipitating event. PTU or methimazole can be used to block hormone synthesis, and iodine or lithium can decrease hormone release. Iodine therapy should be initiated at least one hour after PTU to avoid reflex thyroid hormone release.^6^ In addition, PTU, beta blockers, and glucocorticoids all can help decrease peripheral conversion of T4 to T3. Beta blockers also help decrease the peripheral effects of thyroid hormone.^3^,^9^

Once patients are hemodynamically stable, the mainstay of definitive treatment is evacuation of the mole.^3^ OB evaluation should be obtained early in these patients’ clinical course. Suction and curettage is usually the preferred method of evacuation. Alternatively, hysterectomy is sometimes performed in patients who do not desire further pregnancy.^1^ The evacuation of the mole results in rapid reduction in thyroid hormone levels.^6^ Post-evacuation follow-up with serial quantitative β-hCG measurements is crucial to evaluate for persistent molar tissue or development of choriocarcinoma. These complications develop in about 15–20% of patients with complete mole and 1–5% of patients with partial mole.^1^

## CONCLUSION

GTD-induced thyroid storm is a rare but potentially life-threatening condition. A high level of suspicion and early diagnosis in the ED is critical to obtain appropriate treatment. This diagnosis should be considered in any female patient of childbearing age with signs and symptoms of thyrotoxicosis.

## Figures and Tables

**Image:**
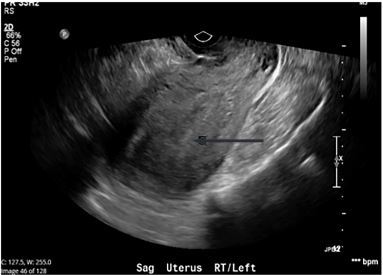
Transvaginal ultrasound showing thickened endometrium (arrow) with no intrauterine pregnancy.

**Table:** Select laboratory values on admission and discharge from hospital.

Test	Admission value	Discharge value	Reference range
WBC	16.1 K/mm^3^	7.4 K/mm^3^	4.0 – 11.0 K/mm^3^
HGB	11.3 g/dL	7.8 g/dL	12.3 – 15.3 g/dL
TSH 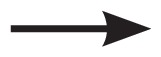	0.009 mIU/mL	0.006 mIU/mL	0.360–3.740 miU/mL
Total T4 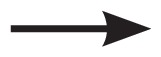	14.7 ug/dL	N/A	4.5 – 10.9 ug/dL
Free T4	1.82 ng/dL	1.75 ng/dL	0.89 – 1.76 ng/dL
Total T3	108.10 ng/dL	N/A	60.00 – 181.00 ng/dL
Quantitative hCG 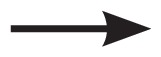	117,495 mIU/mL	44,479 mIU/mL	<5 mIU/mL

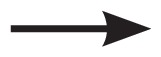
 Decreased TSH and elevated total T4 on admission; 
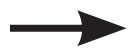
 Elevated quantitative hCG at admission, decreased by time of discharge.

*WBC*, white blood cell count; *HGB*, hemoglobin; *TSH*, thyroid stimulating hormone; *T4*, thyroxine; *T3*, triiodothyronine; *hCG*, human chorionic gonadotropin; *K*, thousand; *mm^3^*, cubic millimeter; *g*, gram; *dL*, deciliter; *mIU*, milli-international unit; *mL*, millileter; *ug*, microgram; *N/A*, not applicable; *ng*, nanogram; *g*, gram.
